# Effectiveness of Monovalent Rotavirus Vaccine in Mozambique, a Country with a High Burden of Chronic Malnutrition

**DOI:** 10.3390/vaccines10030449

**Published:** 2022-03-15

**Authors:** Assucênio Chissaque, Rachel M. Burke, Esperança L. Guimarães, Filomena Manjate, Arsénio Nhacolo, Jorfélia Chilaúle, Benilde Munlela, Percina Chirinda, Jerónimo S. Langa, Idalécia Cossa-Moiane, Elda Anapakala, Adilson Fernando Loforte Bauhofer, Marcelino Garrine, Eva D. João, Júlia Sambo, Luzia Gonçalves, Goitom Weldegebriel, Keith Shaba, Isah Mohammed Bello, Jason M. Mwenda, Umesh D. Parashar, Jacqueline E. Tate, Inácio Mandomando, Nilsa de Deus

**Affiliations:** 1 Instituto Nacional de Saúde (INS), EN1, Bairro da Vila-Parcela nº 3943, Distrito de Marracuene, Maputo 264, Mozambique; esperanca.guimaraes@ins.gov.mz (E.L.G.); jorfelia.chilaule@ins.gov.mz (J.C.); benilde.munlela@ins.gov.mz (B.M.); jeronimo.langa@ins.gov.mz (J.S.L.); idalecia.moiane@ins.gov.mz (I.C.-M.); elda.anapakala@ins.gov.mz (E.A.); adilson.bauhofer@ins.gov.mz (A.F.L.B.); julia.sambo@ins.gov.mz (J.S.); inacio.mandomando@manhica.net (I.M.); nilsa.dedeus@ins.gov.mz (N.d.D.); 2 Instituto de Higiene e Medicina Tropical (IHMT), Universidade Nova de Lisboa, Rua da Junqueira 100, 1349-008 Lisboa, Portugal; filomena.manjate@manhica.net; 3 Centers for Disease Control and Prevention, Atlanta, GA 30329, USA; lxx8@cdc.gov (R.M.B.); uap2@cdc.gov (U.D.P.); jqt8@cdc.gov (J.E.T.); 4 Centro de Investigação em Saúde de Manhiça (CISM), Maputo 1929, Mozambique; arsenio.nhacolo@manhica.net (A.N.); percina.chirinda@manhica.net (P.C.); marcelino.garrine@manhica.net (M.G.); eva.joao@manhica.net (E.D.J.); 5 Institute of Tropical Medicine (ITM), 2000 Antwerp, Belgium; 6 Global Health and Tropical Medicine (GHTM), Instituto de Higiene e Medicina Tropical (IHMT), Universidade Nova de Lisboa (UNL), 1349-008 Lisbon, Portugal; LuziaG@ihmt.unl.pt; 7 Centro de Estatística e Aplicações da Universidade de Lisboa (CEAUL), Faculdade de Ciências da Universidade de Lisboa, 1749-016 Lisbon, Portugal; 8 World Health Organization Regional Office for Africa, Brazzaville P.O. Box 06, Congo; weldegebrielg@who.int (G.W.); shabak@who.int (K.S.); belloi@who.int (I.M.B.); mwendaj@who.int (J.M.M.); 9 Departamento de Ciências Biológicas, Universidade Eduardo Mondlane, Maputo 3453, Mozambique

**Keywords:** children, gastroenteritis, Mozambique, malnutrition, rotavirus, vaccine effectiveness

## Abstract

Mozambique introduced monovalent rotavirus vaccine (Rotarix^®^) in September 2015. We evaluated the effectiveness of Rotarix^®^ under conditions of routine use in Mozambican children hospitalized with acute gastroenteritis (AGE). A test negative case-control analysis was performed on data collected during 2017–2019 from children <5 years old, admitted with AGE in seven sentinel hospital sites in Mozambique. Adjusted VE was calculated for ≥1 dose of vaccine vs. zero doses using unconditional logistic regression, where VE = (1 − aOR) × 100%. VE estimates were stratified by age group, AGE severity, malnutrition, and genotype. Among 689 children eligible for analysis, 23.7% were rotavirus positive (cases) and 76.3% were negative (controls). The adjusted VE of ≥1 dose in children aged 6–11 months was 52.0% (95% CI, −11, 79), and −24.0% (95% CI, −459, 62) among children aged 12–23 months. Estimated VE was lower in stunted than non-stunted children (14% (95% CI, −138, 66) vs. 59% (95% CI, −125, 91)). Rotavirus vaccination appeared moderately effective against rotavirus gastroenteritis hospitalization in young Mozambican children. VE point estimates were lower in older and stunted children, although confidence intervals were wide and overlapped across strata. These findings provide additional evidence for other high-mortality countries considering rotavirus vaccine introduction.

## 1. Introduction

Rotavirus is the most important cause of moderate to severe gastroenteritis in children under five years old [1]. In 2019, about 128,500 deaths by rotavirus were reported worldwide, of which 81.5% (104,733) occurred in sub-Saharan Africa [1]. In Mozambique, the average annual number of deaths due to rotavirus gastroenteritis (RVGE) among children under five from 2017 to 2019 was 1126, and the mortality rate varied from 27 to 19 per 100,000 children [2].

The introduction of rotavirus vaccine in 82 countries contributed to the reduction of rotavirus prevalence among hospitalized paediatric AGE from 38% to 23% between 2008 and 2016 [3]. However, vaccine effectiveness (VE) is lower (<80%) in low-income countries than in developed countries [4], and similarly, vaccine efficacy was also notably lower in clinical trials conducted in this setting [5]; malnutrition is one possible explanation [6]. The burden of malnutrition is high in sub-Saharan African countries [7] and particularly in Mozambique, where more than 40% of children less than five years old suffer from chronic malnutrition [8]. Lower rotavirus VE in malnourished children was observed in studies conducted in African countries such as Botswana, Malawi, Kenya, and Zimbabwe [9,10,11,12]. Therefore, assessing rotavirus vaccine performance in countries with a high burden of malnutrition is important given that this condition may reduce protection in children [6].

As a strategy to estimate the burden of rotavirus in Mozambique, two surveillance systems were implemented in the country. Vigilância Nacional de Diarreias—ViNaDia (National Surveillance of Diarrhoea) was launched by the Instituto Nacional de Saúde (INS) in one hospital in 2014 and extended to five others in 2015, all in urban areas [13]; another diarrhoea surveillance system was established by Centro de Investigação em Saúde de Manhiça (CISM) in September 2015, in the rural district Manhiça.

In September 2015, Mozambique introduced monovalent rotavirus vaccine (Rotarix^®^/GSK, Rixensart, Belgium) through the Expanded Program of Immunization, with doses scheduled at 2 and 3 months of age. Following vaccine introduction, RVGE cases reduced from an average of 39% of paediatric hospitalizations to 13% [13], suggesting that the vaccine has reduced rotavirus disease burden. However, no data are available on the effectiveness of the vaccine in routine use among Mozambican children, which is important to demonstrate the performance of the vaccine in the country. In this analysis, we estimate rotavirus VE against hospitalization in the context of a high burden of malnutrition.

## 2. Materials and Methods

The ViNaDia system was implemented in six urban referral hospitals covering the three regions of the country (southern, central, and northern) in four provinces: Maputo (Hospital Central de Maputo, Hospital Geral de Mavalane, and Hospital Geral José Macamo), Sofala (Hospital Central da Beira), Zambézia (Hospital Geral de Quelimane), and Nampula (Hospital Central de Nampula). ViNaDia’s methodology was previously described elsewhere [14,15]. The CISM diarrhoeal diseases surveillance platform was implemented in Manhiça, a rural area located 80 km north of Maputo province [16], at the Manhiça District Hospital, which is the reference hospital for the whole district; all inclusion criteria and sample collection procedures were based on previously described work [17]. 

### 2.1. Enrolment and Eligibility Criteria 

ViNaDia surveillance included children from 0 to 14 years old (paediatric age in Mozambique), and CISM Surveillance enrolled children 0 to 59 months of age. At both sites, children were recruited if they presented at sentinel sites with diarrhoea, defined as the passage of three or more loose or liquid stools in the last 24 h before seeking healthcare [18]. For eligible children, sociodemographic and clinical data were collected in a structured form by interviewing the parents/caregivers. As confirmation and validation of vaccination status, a copy of each child’s healthcare card was taken. For the present analysis, we included children who were 6–59 months of age, had a valid stool result for rotavirus, were age-eligible to have received rotavirus vaccine (infants born on or after July 2015), did not have chronic or bloody diarrhoea, and had a valid source of vaccination data (health card). Data were restricted to children admitted from January 2017 through December 2019, since vaccine card capture was inconsistent before this period.

### 2.2. Sample Collection and Laboratory Procedure 

At least one stool specimen (10 mL) was collected for each eligible child and sent to the INS (samples from ViNaDia) or CISM (from Diarrhoeal Diseases Surveillance) laboratory for rotavirus diagnosis using the commercial enzyme-immunoassay (ELISA) kit ProSpecT Rotavirus Microplate Assay (Oxoid Ltd., Basingstok, UK). Samples were tested following the manufacturer’s instructions, and all positive samples were genotyped using procedures that were previously described [19,20,21].

### 2.3. Exposure and Outcomes Definition

The controls were children with acute diarrhoea who tested negative for rotavirus, while the cases were children that tested positive. The vaccination status was defined as unvaccinated (children that received zero doses of Rotarix vaccine), partially vaccinated (children that received one dose of Rotarix), or fully vaccinated (children that received two doses of Rotarix). Each dose had to be administered at least 14 days prior to admission to count towards vaccination status. Vaccination data were obtained from the child’s vaccination card, and children without any acceptable vaccination record (no record or only maternal report) or with an incomplete vaccination record (record available but rotavirus status marked as unknown) were excluded. The control group was used to estimate rotavirus vaccination coverage among the target population.

### 2.4. Statistical Analysis 

Data analysis was performed using R version 4.0.5. Chi-square, Fisher’s exact (categorical variables), and Mann–Witney U tests (ordinal or continuous variables) were used to compare sociodemographic and clinical characteristics between cases and controls. Unconditional simple and multiple logistic regression models were used to estimate crude and adjusted VE, respectively, as (1 − aOR) × 100%, where vaccination status was the exposure. Models were adjusted for child age, rural location of hospital, and season of admission (January–June versus July–December). These potential confounders were chosen a priori based on literature review. Vaccination status was dichotomized as any doses (partially or fully vaccinated) or zero doses (unvaccinated), due to low sample size. Wald 95% confidence intervals (CI) are presented for all VE estimates. A *p*-value < 0.05 was considered significant.

### 2.5. Definitions of Covariates

Diarrhoea severity was estimated by using the 20-point clinical modified Vesikari score, dichotomized into less severe <11 and severe ≥11 (Supplementary Table S1). The nutritional status was calculated using WHO software Anthro version 3.2.2. Height-for-age Z-score (HAZ) was used to classify children as non-stunted (−2 ≤ HAZ ≤ +2) or stunted (−6 ≤ HAZ < −2) [22]. The genotype used for stratification in this analysis was G1P[8], which is a commonly circulating genotype and also the genotype of the Rotarix vaccine.

### 2.6. Ethical Statement

The ViNaDia protocol was approved by the National Health Bioethics Committee of Mozambique (CNBS) under number IRB00002657, reference number: 348/CNBS/13. The CISM Diarrhoeal Disease Surveillance platform was approved by the same Committee under the reference number 209/CNBS/15; IRB00002657.

## 3. Results

**Figure 1:**
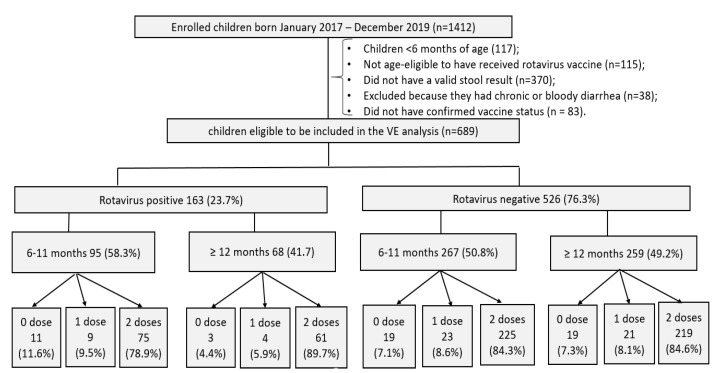
Flowchart of enrolled children included in rotavirus VE analysis by age, 2017–2019.

From January 2017 through December 2019, about 1412 children with acute diarrhoea were enrolled in the two surveillance systems. A total of 689 (48.8%) children aged between 6 and 59 months hospitalized with AGE met the inclusion criteria for VE analysis, with the majority from urban hospitals (84.5%; 582/689) and 15.5% (107/689) from a rural hospital. Overall, 23.7% (163/689) of children were positive for rotavirus (cases), and 76.3% (526/689) were negative (controls) (Figure 1).

The largest number of children in the analysis was enrolled in 2017 (Table 1), and the distribution of enrolment by year differed between cases and controls (*p*-value = 0.003). Rotavirus infection was higher in male children (55.2% cases, and 44.8% controls were male). Children in the case group tended to live in larger households than children in the control group (median five (cases) vs. four members (controls), *p*-value < 0.001), and a similar pattern was seen for the number of children in the home (two for cases vs. one for controls, *p*-value = 0.002).

There were similar proportions of stunted children (cases 36.1% and controls 37.2%) (Table 1). Most of the stunted children (48.4%) were enrolled from Hospital Central de Nampula in northern Mozambique (Supplementary Table S2).

There was a significant geographical difference in distribution of rotavirus (*p*-value < 0.001). Regarding the economic factors, children in the case group were less likely to live in households with electricity, refrigerator, or cell phone than children in the control group (Table 1).

### 3.1. Rotavirus Vaccine Coverage

Rotavirus vaccination among eligible children was high (84.4%) among controls, and most of the children received the first and second doses within 1 month of the recommended age (2 and 3 months, respectively) (Supplementary Figure S1).

### 3.2. Vaccine Effectiveness

The adjusted effectiveness of at least one dose of rotavirus vaccine against RVGE hospitalization in all age groups was 35% (95% CI, −30, 66). Among children <12 months of age, adjusted VE was 52% (95% CI, −11, 79), and among children from 12 to 23 months, it was −24% (95% CI, −459, 62). VE was not calculated in children between 24 and 59 months due to low sample size (Table 2).

Stratifying within the first year of age of children, a higher VE was observed in children 6 to 8 months of age (56% [95% CI −65, 85]), with a reduction in children aged 9 to 11 months (46% [95% CI, −70, 82]). The estimate of VE against more severe rotavirus infection was 54% (95% CI, −58, 87), and for less severe, lower at −18% (95% CI, −510, 71). Regarding nutritional status, rotavirus VE was higher in children not stunted (59% [95% CI, −125, 91]), and notably lower among stunted children (14% [95% CI, −138, 66]). VE stratified by genotype showed a 30% (95% CI, −355, 81) for G1P[8] genotype and 35% (95% CI, −35, 67) for non-G1P[8] rotavirus genotype (Table 2). Analyses of VE for full and partial vaccination are presented as Supplementary Table S3, again stratified by age group, severity status, malnutrition, and genotype.

## 4. Discussion

The present analysis describes the “real-world” VE of monovalent rotavirus vaccine in Mozambican children hospitalized with AGE during 2017–2019. In this setting with a high burden of chronic malnutrition, the adjusted estimate of VE for at least one dose of rotavirus vaccine against admission for RVGE was 35% (95% CI, −30, 66) in children from 6 to 59 months of age. The estimated VE was higher, 52% (95% CI, −11, 79), in children from 6 to 11 months of age, which, although non-significant, is similar to the point estimates reported in other low-income countries with high child mortality: 52–86% with a median of 58% [4]. Our VE point estimate is very similar to that reported in Botswana, which was the lowest in the region at 52%, followed by neighbouring countries such as South Africa (54%), Tanzania (56%), and Zimbabwe (61%) [9,10,23,24]. In contrast, rotavirus VE in high-income countries is higher (83–91%) [4]. Similar discrepancies were reported for results from randomized clinical trials conducted in low- as compared with high-income countries. Rotarix vaccine clinical trials conducted in low-income countries reported a lower vaccine efficacy in countries such as Malawi (49.4%) and South Africa (76.9%) [25], and the same was seen in a clinical trial conducted in Ghana (55.5%), Kenya (63.9%), and Mali (17.6%) using pentavalent rotavirus vaccine [26]. In contrast, trials in high-income, low child mortality countries mostly found vaccine efficacy to be >90% [5].

The lower estimates in low-income countries may be due to factors such as greater and earlier exposure to the risk of rotavirus infection, early and co-infection with other enteropathogens [27], interference caused by other vaccines (such as oral polio vaccine) [28], nutritional deficiency [6], interference from maternal antibodies [29], genetic diversity of the virus [30], and others.

A stratified analysis of VE by age showed a higher protection in children from 6 to 11 months of age (52% (95% CI, −11, 79)), compared with older children ≥12 months of age (−24.0% (95% CI, −459, 62)). Lower VE in children ≥12 months were also found in 12 studies conducted in countries with medium and high mortality [31]. Our findings may be due to the waning of protection of rotavirus vaccine [32], but we cannot rule out the major limitation of small sample size and the resulting lower power, which might have contributed to the lower VE and large confidence intervals in this age group. As a strategy to improve VE in low income countries, an additional dose has been suggested for children at 9 months of age [27]; however, a recent analysis hypothesized that if the first two doses failed to generate an immune response, the third or additional will also probably fail [33]. On the other hand, a systematic review found that vaccines given in two and three doses confer a similar level of protection at the final dose. However, since the last dose of a two-dose vaccine is given earlier, at 4 months, the decline in protection begins earlier than with three-dose vaccines, which have the last dose given at 6 months of age [32]. Further studies are needed to better understand the reasons for lower VE in older children, particularly those living in high-mortality countries. This is important since if the vaccine has high effectiveness in the youngest ages, then a shift in the age distribution of RVGE may occur.

The estimate of VE against more severe rotavirus infection was higher compared with less severe infection, 54% versus −24%, respectively. Although CI were wide and overlapping, this finding suggests that rotavirus vaccine may be most impactful in preventing severe infection in Mozambican children. This result is similar to that reported in nine countries with medium- and high-mortality, where the median VE among children with Vesikari score ≥ 11 points was 54% [31]. Our results should be interpreted with caution due to wide and overlapping CI, and we cannot rule out the possibility that this was a chance finding. Additionally, the especially wide CI around the VE estimate for less-severe AGE make that result difficult to interpret.

In Mozambique, more than 40% of children less than five years old suffer from chronic malnutrition [8]. Malnutrition may cause immune deficits and increase the risk of severe infections, thus affecting the vaccine efficacy and effectiveness in children from poor settings [34,35]. Lower rotavirus VE in malnourished children was observed in studies conducted in African countries such as Botswana, Malawi, Kenya, and Zimbabwe [9,10,11,12]. In our data, nutritional stratification analysis of VE showed a much higher protection for well-nourished children (59%) versus stunted children (14%), although confidence intervals overlapped due to small sample size. Similar differences in well-nourished versus stunted children were found in some African countries, such as Botswana (72% vs. −20% for at least one dose), Malawi (78.1% vs. 27.8% for two doses of vaccine), and Zimbabwe (71% vs. 45% in children < 12 months) [9,10,11,12]. Patterns from an analysis in Kenya were less clear: non-stunted children showed the highest VE (75%), and children that presented with moderate-to-severe stunting showed VE of 28%, but VE among the subset of children with severe stunting showed a VE of 69% [12]; given the wide and overlapping confidence intervals and the very small number of severely stunted rotavirus cases, this unexpected trend may have been due to chance. Despite small sample size and limited statistical power, the lower VE point estimate in stunted children in the present study is informative and suggests that interventions to improve nutritional status and immunization coverage may be beneficial. Indeed, prior evidence suggests that interventions in the areas of nutrition and immunization may have synergistic effects. Evidence from Bangladesh and Zambia found that zinc combined with vaccination reduced the risk of rotavirus diarrhoea [36,37].

The low estimate of overall VE in Mozambique compared with other countries may be related to the fact that Mozambique has the highest prevalence of chronic malnutrition (>40%) in children less than 5 years old compared with other African countries (27.0–34.0%), according to the latest community surveys of each country [8,38,39,40]. The burden of malnutrition in Mozambique may be concentrated in specific geographies. Almost half of stunted children of this study were admitted to Hospital Central de Nampula (48.4%, Supplementary Table S2), a poor resource setting in northern Mozambique. Our previous analysis reported that the Hospital Central de Maputo and Hospital Central de Nampula had a high frequency of children with a “triple condition” (undernutrition, HIV, and rotavirus infection) [41]. Another study conducted in Manhiça, a rural area, identified malnutrition as one of the risk factors for death in children with diarrhoea [42].

Rotavirus G1P[8] was the most common circulating genotype during the study period, and this is the principal component of the monovalent vaccine Rotarix. In the present analysis, the VE was similar for G1P[8] (30%) and non-G1P[8] (35%) rotavirus genotypes, which is not concordant with results reported in Kenya in which they found 60% VE against G1P[8] and only 31% for G2P[4] [12]. Other previous studies have also demonstrated that the rotavirus VE against vaccine strains is somewhat higher in comparison to that against nonvaccine strains; however, limited data are available in low-income countries [30]. The findings of the present analysis should be interpreted with caution, as the sample size was small, which limited power to detect differences between VE against G1P[8] and non-G1P[8] rotavirus genotypes. The non-G1P[8] corresponded to 85.1% (131/154) of the genotypes, and the five most-common were G3P[4], G3P[8], G9P[4], G9P[6], and G9P[8]. Other genotypes observed in lower frequency included G2P[4], G1G3P[8], G8P[4], G12P[4], G12P[8], G1P[4], G3P[6], and G4P[4]. Partly non-typeable genotyped samples included G3P[NT], G9P[NT], GNTP[10], GNTP[4], GNTP[6], and GNTP[8].

The present analysis had some limitations. A major limitation was a low sample size combined with high vaccination coverage, which limited statistical power, especially in children ≥12 months, and made it difficult to control potential confounders such as markers of socioeconomic status. Achieved statistical power to detect a VE of 50% was <80% for our sample size, and lower for stratified analyses. For this reason, it is not surprising that none of the estimates of VE were statistically significant, and that confidence intervals were wide. We also had to exclude 83 children from the analysis because they did not have confirmed vaccination status. Our inability to include data from immediately following vaccine introduction (i.e., data from 2016), due to the inconsistent collection of vaccination cards, was another limitation. However, a strength of this analysis is the breadth of data available, which allowed us to stratify VE estimates by many characteristics of interest, to identify factors that may play a role in the lower VE estimate in the country. Additionally, although estimates were imprecise, the general findings and patterns are in line with previous research, suggesting that our conclusions are reasonable despite the limitations of the data.

In conclusion, at least one dose of rotavirus vaccine was estimated to have 52% effectiveness against rotavirus admissions among children aged 6–11 months old in Mozambique, a country with a high burden of chronic malnutrition. The VE estimate from Mozambique is lower than that seen in many other African countries, possibly due to the high burden of chronic malnutrition in children under five years old. This analysis was not powered to detect statistical differences in VE stratified by nutritional status, although stunting appeared to diminish VE. It would be important to understand the role of stunting in vaccine efficacy/effectiveness in Mozambican children using studies with appropriate design and adequate sample size. Despite our inability to detect significant differences, our findings are generally in line with other studies showing the benefits of rotavirus vaccination under conditions of “real-world” usage.

## Figures and Tables

**Table 1:** Vaccination, anthropometric and sociodemographic characteristics of cases and controls, Mozambique 2017–2019.

Characteristic	Cases (*n* = 163)	Controls (*n* = 526)	*p* Value *
**RV Vaccination Status**			
Unvaccinated	14 (8.6%)	38 (7.2%)	0.84
Partially Vaccinated	13 (8%)	44 (8.4%)	-
Fully Vaccinated	136 (83.4%)	444 (84.4%)	-
**Year of Admission**			
2017	91 (55.8%)	216 (41.1%)	0.003
2018	40 (24.5%)	193 (36.7%)	-
2019	32 (19.6%)	117 (22.2%)	-
**Sex**			
Female	73 (44.8%)	196 (37.3%)	0.10
Male	90 (55.2%)	330 (62.7%)	-
**Age Group**			
6–8 months	42 (25.8%)	114 (21.7%)	0.45
9–11 months	53 (32.5%)	153 (29.1%)	-
12–17 months	43 (26.4%)	153 (29.1%)	-
18–23 months	17 (10.4%)	59 (11.2%)	-
24–35 months	6 (3.7%)	40 (7.6%)	-
36–47 months	2 (1.2%)	7 (1.3%)	-
**Number Persons in HH (median)**	5.0 (4.0, 7.0)	4.0 (3.0, 6.0)	0.001
**Number Children in HH (median)**	2.0 (1.0, 2.0)	1.0 (1.0, 2.0)	0.003
**Anthropometrics**			
Normal height for age	75 (63.6%)	234 (63.8%)	1.00
Stunted (HAZ < −2)	43 (36.4%)	133 (36.2%)	-
Missing	45	159	-
**Surveillance Site**			<0.001
Centro de Saude da Manhica	27 (16.6%)	80 (15.2%)	
Hospital Central da Beira	5 (3.1%)	34 (6.5%)	-
Hospital Central de Maputo	13 (8.0%)	79 (15.0%)	-
Hospital Central de Nampula	59 (36.2%)	112 (21.3%)	-
Hospital Gera Jose Macamo	17 (10.4%)	59 (11.2%)	-
Hospital Geral de Mavalane	25 (15.3%)	137 (26.0%)	-
Hospital Geral de Qualimane	17 (10.4%)	25 (4.8%)	-
**Electricity**			
Yes	108 (66.7%)	422 (80.7%)	<0.001
No	54 (33.3%)	101 (19.3%)	-
Unknown	1	3	-
**House Type**			
Reed house	12 (7.4%)	31 (6%)	<0.001
Mud house	57 (35%)	96 (18.4%)	-
Brick house	94 (57.7%)	394 (75.6%)	-
Missing	0	5	-
**Fridge**			
Yes	67 (41.1%)	286 (54.5%)	0.004
No	96 (58.9%)	239 (45.5%)	-
Unknown	0	1	-
**Cell Phone**			
Yes	124 (76.5%)	445 (84.8%)	0.021
No	38 (23.5%)	80 (15.2%)	-
Unknown	1	1	-

* *p*-values calculated using Mann–Whitney U for number of people or children in households and Chi-Squared or Fisher’s Exact (for expected cell size < 5) for all categorical variables. Bold: overall characteristic measured, with response options immediately below.

**Table 2:** Vaccine effectiveness estimates by different characteristics of the children, 2017–2019.

Model	Cases	Controls	Crude			Adjusted *		
	Vaccinated/Total (%)	Vaccinated/Total (%)	VE	95% CI	*p*-Value	VE	95% CI	*p*-Value
6 to 59 months	148/162 (91.4%)	488/526 (92.8%)	18	(−61, 56)	0.55	35	(−30, 66)	0.20
6 to 8 months	37/42 (88.1%)	107/114 (93.9%)	52	(−72, 85)	0.24	56	(−65, 88)	0.21
9 to 11 months	47/53 (88.7%)	141/153 (92.2%)	33	(−100, 76)	0.44	46	(−70, 82)	0.27
6 to 11 months	84/95 (88.4%)	248/267 (92.9%)	41	(−32, 73)	0.18	52	(−11, 79)	0.078
12 to 23 months	56/59 (94.9%)	195/212 (92%)	−63	(−615, 48)	0.45	−24	(−459, 62)	0.74
Severe: Modified Vesikari Score ≥ 11	51/58 (87.9%)	73/81 (90.1%)	20	(−141, 73)	0.68	54	(−58, 87)	0.21
Less severe: Modified Vesikari Score < 11	25/28 (89.3%)	69/78 (88.5%)	−9	(−419, 70)	0.91	−18	(−510, 71)	0.82
Stunted (HAZ < −2)	36/43 (83.7%)	114/133 (85.7%)	14	(−134, 65)	0.75	14	(−138, 66)	0.76
Not Stunted (HAZ ≥ −2)	72/75 (96%)	226/234 (96.6%)	15	(−296, 76)	0.81	59	(−125, 91)	0.27
G1P[8] Rotavirus	21/23 (91.3%)	488/526 (92.8%)	18	(−423, 77)	0.79	30	(−355, 81)	0.64
Non-G1P[8] Rotavirus #	127/139 (91.4%)	488/526 (92.8%)	18	(−69, 57)	0.58	35	(−35, 67)	0.22

* Adjusted for age, rural location of hospital, and season of admission (January–June vs. July–December). # The five most-common non-G1P[8] genotypes with high frequency were: G3P[4] (19.1%), G3P[8] (16.8%), G9P[4] (14.5%), G9P[6] (9.9%), and G9P[8] (8.4%). Other genotypes observed with lower frequency included G2P[4] (6.9%), G1G3P[8] (3.1%), G8P[4] (2.3%), G12P[4] (0.8%), G12P[8] (0.8%), G1P[4] (0.8%), G3P[6] (0.8%), and G4P[4] (0.8%). Partially non-typeable genotyped samples included GNTP[4] (7.6%), G3P[NT] (1.5%), GNTP[8] (3.1%), GNTP[6] (1.5%), G9P[NT] (0.8%), and GNTP[10] (0.8%).

## Data Availability

The data are available upon reasonable request from the corresponding author.

## Supplementary Materials

The following supporting information can be downloaded at https://www.mdpi.com/article/10.3390/vaccines10030449/s1, Figure S1: Cumulative percentage of vaccinated children by age (in weeks); Table S1: Modified 20-point clinical modified Vesikari score; Table S2: Distribution of stunted children by sentinel sites (2017–2019); Table S3: Vaccine effectiveness estimates by different characteristics of the children in fully vaccinated vs. unvaccinated and partially vaccinated vs. unvaccinated children, 2017–2019.
