# Supplementary material for: Effectiveness of Monovalent Rotavirus Vaccine in Mozambique, a Country with a High Burden of Chronic Malnutrition
Source: Vaccines (Basel). 2022 Mar 15;10(3):449. doi: 10.3390/vaccines10030449 (PMC8953339; doi:10.3390/vaccines10030449)
Supplement: Supplementary file 1 [file vaccines-10-00449-s001.zip › Supplementary Table S1.pdf]

Supplementary Table S1: Modified 20-point clinical modified Vesikari score.

Symptom or Sign	Vesikari 20 points scale	Mozambique Modification	Notes
Duration of diarrhea			
1 - 4 days	1	1	*24 hours prior the interview*
5 days	2	2	
≥ 6 days	3	3	
Max stools/24h			
1-3	1	1	*24 hours prior the interview*
4-5	2	2	
≥ 6	3	3	
Duration of vomiting			
1 day	1	1	*24 hours prior the interview*
2 days	2	2	
≥ 3 days	3	3	
Max vomiting / 24h			
0	0	0	
1	1	1	*24 hours prior the interview*
2 - 4	2	2	
≥5	3	3	
Fever (º Celsius)			
< 37.0	0	0	
37.1 - 38.4	1	1	*24 hours prior the interview*
38.5 - 38.9	2	2	
≥ 39	3	3	
Dehydration			
None	0	0	*None*
1 - 5%	2	2	*Mild / Moderate*
≥ 6%	3	3	*Severe*
Treatment			
None	0	0	
Rehydration	1	2	*IV Rehydration*
Hospitalization	2	*NA	*All children are hospitalized*

NA: Non Applicable

Tarja Ruuska & Timo Vesikari (1990) Rotavirus Disease in Finnish Children: Use of Numerical Scores for Clinical Severity of Diarrhoeal Episodes, Scandinavian Journal of Infectious Diseases, 22:3, 259-267, DOI: 10.3109/00365549009027046
