# Supplementary material for: Effectiveness of Monovalent Rotavirus Vaccine in Mozambique, a Country with a High Burden of Chronic Malnutrition
Source: Vaccines (Basel). 2022 Mar 15;10(3):449. doi: 10.3390/vaccines10030449 (PMC8953339; doi:10.3390/vaccines10030449)
Supplement: Supplementary file 1 [file vaccines-10-00449-s001.zip › Supplementary Table S2.pdf]

Supplementary Table S2. Distribution of stunted children by sentinel sites (2017-2019).

**Site name**	**Stunting**		**Total (%)**
	**No (%)**	**Yes (%)**	
Centro De Saúde Da Manhiça	75 (23.9)	**30 (16.3)**	105 (21.1)
Hospital Central Da Beira	18 (5.7)	8 (4.3)	26 (5.2)
Hospital Central De Maputo	20 (6.4)	4 (2.2)	24 (4.8)
Hospital Central De Nampula	54 (17.2)	**89 (48.4)**	143 (28.7)
Hospital Gera Jose Macamo	37 (11.8)	14 (7.6)	51 (10.2)
Hospital Geral De Mavalane	106 (33.8)	**35 (19.0)**	141 (28.3)
Hospital Provincial De Quelimane	4 (1.3)	4 (2.2)	8 (1.6)
**Total**	**314**	**184**	**498**
