# Supplementary material for: Effectiveness of Monovalent Rotavirus Vaccine in Mozambique, a Country with a High Burden of Chronic Malnutrition
Source: Vaccines (Basel). 2022 Mar 15;10(3):449. doi: 10.3390/vaccines10030449 (PMC8953339; doi:10.3390/vaccines10030449)
Supplement: Supplementary file 1 [file vaccines-10-00449-s001.zip › Supplementary Table S3.pdf]

Supplementary Table S3. Vaccine effectiveness estimates by different characteristics of the children in fully vaccinated vs unvaccinated &Partially vaccinated vs unvaccinated children 2017-2019.

Model	Controls (vaccinated/total)	Cases (vaccinated/total)	Crude			Adjusted*		
			VE	95% CI	P-value	VE	95% CI	P-value
Overall								
Partially vaccinated	44/526 (8.4%)	13/163 (8%)	20	(-92, 67)	0.62	35	(-60, 74)	0.35
Fully vaccinated	444/526 (84.4%)	136/163 (83.4%)	17	(-63, 55)	0.57	35	(-31, 66)	0.21
6 to 8-month-olds only								
Partially vaccinated	8/114 (7%)	5/42 (11.9%)	13	(-343, 83)	0.87	15	(-390, 85)	0.85
Fully vaccinated	99/114 (86.8%)	32/42 (76.2%)	55	(-62, 87)	0.20	60	(-54, 89)	0.17
9 to 11-month-olds only								
Partially vaccinated	15/153 (9.8%)	4/53 (7.5%)	47	(-130, 89)	0.40	52	(-121, 90)	0.35
Fully vaccinated	126/153 (82.4%)	43/53 (81.1%)	32	(-106, 75)	0.47	45	(-73, 81)	0.28
6 to 11 month-olds only								
Partially vaccinated	23/267 (8.6%)	9/95 (9.5%)	32	(-97, 77)	0.47	40	(-83, 81)	0.37
Fully vaccinated	225/267 (84.3%)	75/95 (78.9%)	42	(-30, 73)	0.17	54	(-9, 80)	0.07
12 to 23-month-olds only								
Partially vaccinated	19/212 (9%)	3/60 (5%)	11	(-441, 85)	0.90	31	(-331, 89)	0.68
Fully vaccinated	176/212 (83%)	54/60 (90%)	-74	(-665, 44)	0.39	-33	(-498, 59)	0.67
Severe: Modified Vesikari Score >=11								
Partially vaccinated	11/81 (13.6%)	8/58 (13.8%)	17	(-228, 79)	0.79	56	(-101, 91)	0.30
Fully vaccinated	62/81 (76.5%)	43/58 (74.1%)	21	(-142, 73)	0.68	54	(-61, 87)	0.22
Less severe: Modified Vesikari Score < 11								
Partially vaccinated	10/78 (12.8%)	2/28 (7.1%)	40	(-344, 93)	0.62	32	(-424, 93)	0.71
Fully vaccinated	59/78 (75.6%)	23/28 (82.1%)	-17	(-462, 68)	0.83	-34	(-604, 69)	0.70
Stunted (HAZ < -2)								
Partially vaccinated	13/133 (9.8%)	3/43 (7%)	37	(-172, 88)	0.55	37	(-175, 88)	0.55
Fully vaccinated	101/133 (75.9%)	33/43 (76.7%)	11	(-144, 65)	0.80	10	(-151, 65)	0.83

Not Stunted (HAZ >= -2)								
Partially vaccinated	20/234 (8.5%)	8/75 (10.7%)	-7	(-480, 76)	0.94	49	(-222, 91)	0.45
Fully vaccinated	206/234 (88%)	64/75 (85.3%)	17	(-287, 77)	0.79	60	(-120, 91)	0.26
G1P[8] Rotavirus								
Partially vaccinated	44/526 (8.4%)	1/23 (4.3%)	57	(-368, 98)	0.50	62	(-324, 98)	0.44
Fully vaccinated	444/526 (84.4%)	20/23 (87%)	14	(-449, 76)	0.84	27	(-376, 81)	0.68
Non-G1P[8] Rotavirus								
Partially vaccinated	44/526 (8.4%)	12/140 (8.6%)	14	(-116, 66)	0.75	29	(-81, 72)	0.47
Fully vaccinated	444/526 (84.4%)	116/140 (82.9%)	17	(-70, 57)	0.59	36	(-35, 67)	0.22

Unconditional logistic regression. *Adjusted for age, rural location of hospital, and season of admission (Jan-June vs. July- Dec).
