# Supplementary figures and images for: Effectiveness of Monovalent Rotavirus Vaccine in Mozambique, a Country with a High Burden of Chronic Malnutrition
Source: Vaccines (Basel). 2022 Mar 15;10(3):449. doi: 10.3390/vaccines10030449 (PMC8953339; doi:10.3390/vaccines10030449)

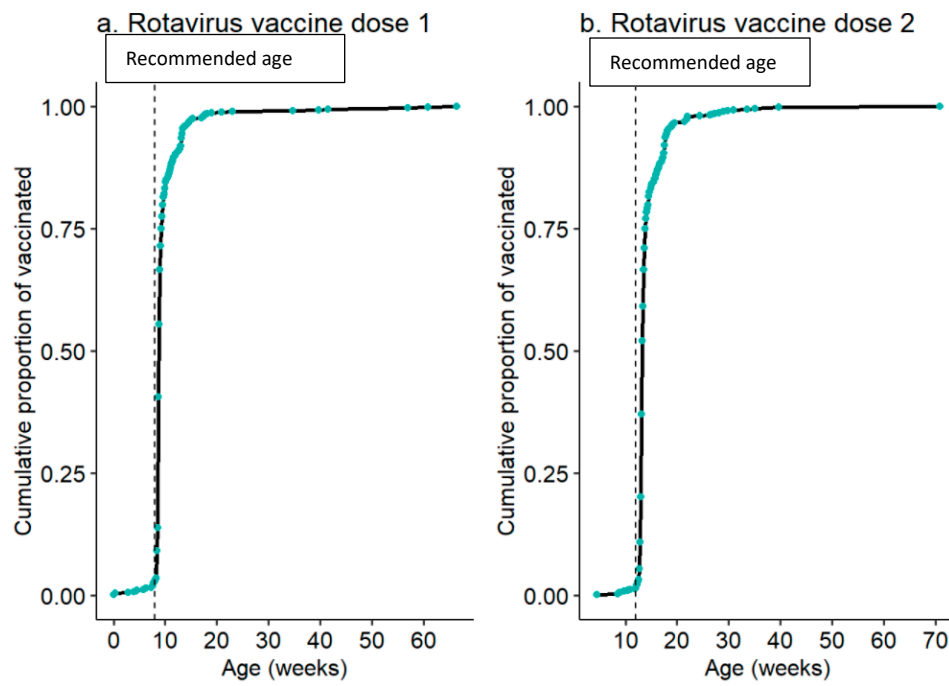

**Supplementary Figure S1.** Cumulative percentage of vaccinated children by age (in weeks).

Supplement: Supplementary file 1 [file vaccines-10-00449-s001.zip › Supplementary Figure S1.pdf]
